# (*Z*)-Ethyl 2-cyano-3-(1*H*-imidazol-2-yl)acrylate

**DOI:** 10.1107/S1600536813018278

**Published:** 2013-07-10

**Authors:** Rajesh G. Kalkhambkar, Mahesh Kumar, D. Gayathri, Jeongsu Oh, Yeon Tae Jeong

**Affiliations:** a Department of Chemistry, Karnatak University’s Karnatak Science College, Dharwad 580 001, Karnatak, India; b Department of Biotechnology, Dr. M.G.R Educational and Research Institute University, Periyar E.V.R. High Road, Maduravoyal, Chennai 600 095, India; c Department of Image Science and Engineering, Pukyong National University, Busan 608 739, Republic of Korea

## Abstract

The crystal structure of the title compound, C_9_H_9_N_3_O_2_, features N—H⋯N and C—H⋯O inter­actions. The N—H⋯N inter­action generates a chain running along the *a* axis and the C—H⋯O inter­action generates a chain along the *c* axis. An intra­molecular C—H⋯O inter­action is also observed.

## Related literature

For background references and the biological importance of related compounds, see: Bigi *et al.* (1999 ▶); Yu *et al.* (2000 ▶). For the synthesis, see: Knoevenagel (1898 ▶); Yadav *et al.* (2004 ▶). 
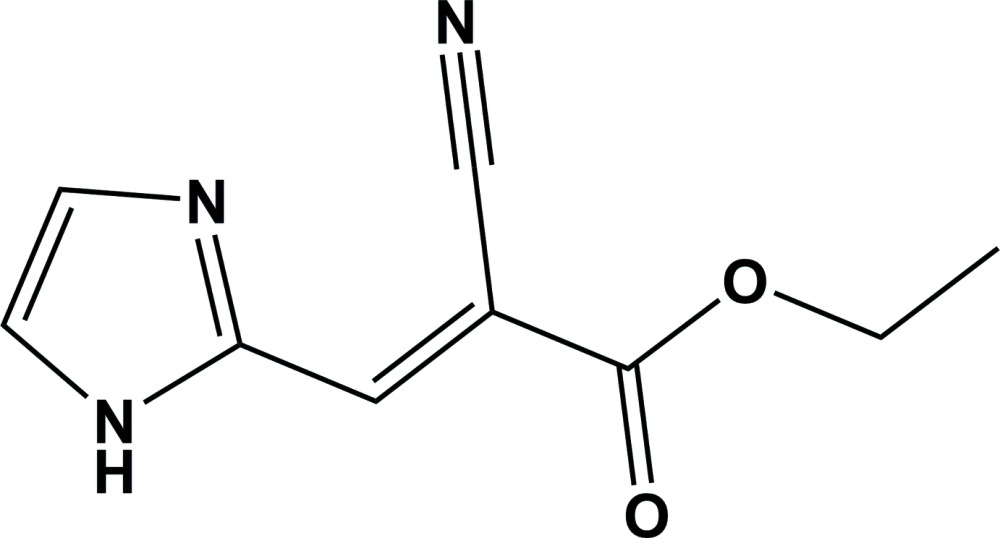


## Experimental

### 

#### Crystal data

C_9_H_9_N_3_O_2_
*M* *_r_* = 191.19
Orthorhombic, 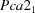

*a* = 10.0768 (7) Å
*b* = 12.0387 (8) Å
*c* = 7.7047 (6) Å
*V* = 934.67 (12) Å^3^
*Z* = 4
Mo *K*α radiation
μ = 0.10 mm^−1^
*T* = 293 K
0.4 × 0.23 × 0.2 mm

#### Data collection

Bruker SMART CCD area-detector diffractometer
Absorption correction: multi-scan (*SADABS*; Bruker, 2001 ▶) *T*_min_ = 0.980, *T*_max_ = 0.984
7118 measured reflections
1436 independent reflections
1189 reflections with ( > 2σ(*I*)
*R*_int_ = 0.033

#### Refinement

*R*[*F* ^2^ > 2σ(*F* ^2^)] = 0.049
*wR*(*F* ^2^) = 0.145
*S* = 0.83
1436 reflections
128 parameters
1 restraint
H-atom parameters constrained
Δρ_max_ = 0.33 e Å^−3^
Δρ_min_ = −0.22 e Å^−3^

### 

Data collection: *SMART* (Bruker 2001 ▶); cell refinement: *SAINT* (Bruker, 2001 ▶); data reduction: *SAINT*; program(s) used to solve structure: *SHELXS97* (Sheldrick, 2008 ▶); program(s) used to refine structure: *SHELXL97* (Sheldrick, 2008 ▶); molecular graphics: *PLATON* (Spek, 2009 ▶); software used to prepare material for publication: *SHELXL97*.

## Supplementary Material

Crystal structure: contains datablock(s) I, global. DOI: 10.1107/S1600536813018278/fj2635sup1.cif

Structure factors: contains datablock(s) I. DOI: 10.1107/S1600536813018278/fj2635Isup2.hkl

Supplementary material file. DOI: 10.1107/S1600536813018278/fj2635Isup3.cml

Additional supplementary materials:  crystallographic information; 3D view; checkCIF report

## Figures and Tables

**Table 1:** Hydrogen-bond geometry (Å, °)

*D*—H⋯*A*	*D*—H	H⋯*A*	*D*⋯*A*	*D*—H⋯*A*
C4—H4⋯O1	0.93	2.38	2.771 (3)	105
N1—H1*A*⋯N2^i^	0.86	2.11	2.951 (3)	167
C1—H1⋯O1^ii^	0.93	2.45	3.328 (4)	158

Symmetry codes: (i) 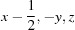; (ii) 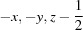.

**Fig. 1.**
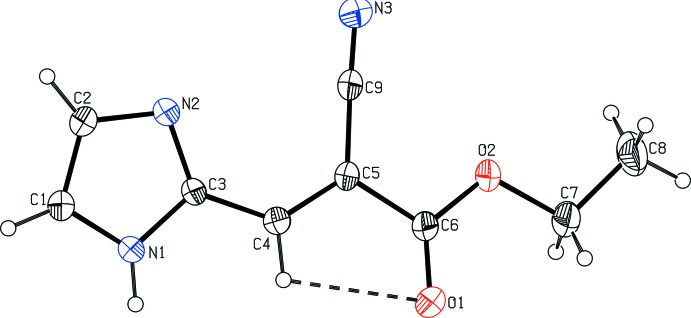
The molecular structure of title compound, showing 30% probability displacement ellipsoids.

**Fig. 2.**
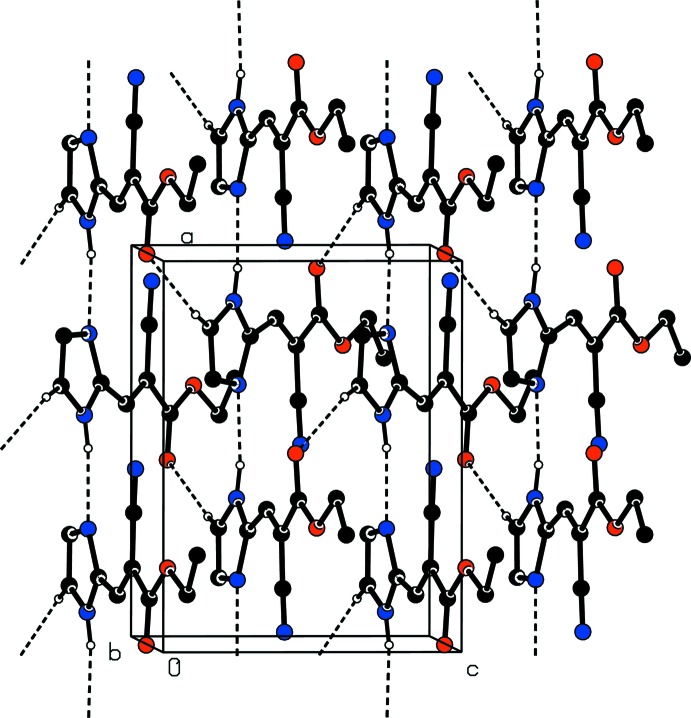
The molecular packing of (I). For clarity, hydrogen atoms which are not involved in hydrogen bonding are omitted.

## supplementary crystallographic information

### Comment

The Knoevenagel condensation is an important carbon-carbon bond-forming reaction in organic synthesis (Knoevenagel, 1898; Yadav *et al.*, 2004). This reaction has been widely used in organic synthesis to prepare many biologically active derivatives, and in the synthesis of cosmetics, perfumes and pharmaceuticals (Bigi *et al.*, 1999; Yu *et al.*, 2000). With the view of biological importance, the series of compounds are synthesized and here we report the crystal structure of the title compound.

The bond lengths and bond angles are within the normal ranges. The imidazole ring is planar and the acrylate moiety is nearly planar with torsion angles C4—C5—C6—O1 and C4—C5—C6—O2 being -6.1 (5) and 174.2 (3)°, respectively. Atoms C6, C9, N3 and O1 deviate from the planarity of the title compound with maximum deviation of 0.31 (3) Å by O1 atom. The crystal structure is stabilized by C(4)—H(4)···O1 intramolecuar interaction generating S(5) motif. The crystal packing is stabilized by N—H···N and C—H···O intermolecular interaction network. Atom N1 acts as a donor to N2 generating chain of C(4) along *a* axis and atom C1 acts as a donor to O1 generating C(8) chain running along *c* axis.

### Experimental

A solution of 1*H*-Imidazole-2-aldehyde (1 mol), ethyl cyanoacetate (1.2 mol) and piperidine (0.1 ml) in ethanol (20 ml) was stirred at room temperature for 4 h. After removal of the volatiles *in vacuo*, yellow solid was obtained in quantitative yield. A sample for analysis was obtained by recrystallization from EtOAc as pale yellow needles.

### Refinement

Friedel pairs were merged in the absence of any anamalous scatterers in the molecule. The absolute structure in the present model has been chosen arbitrarily. All H-atoms were refined using a riding model with d(C—H) = 0.93 Å, *U*_iso_ = 1.2*U*_eq_ (C) for aromatic, 0.97 Å, *U*_iso_ = 1.2*U*_eq_ (C) for CH_2_ and 0.96 Å, *U*_iso_ = 1.5*U*_eq_ (C) for CH_3_ atoms

### Crystal data

**Table:**

C_9_H_9_N_3_O_2_	*F*(000) = 400
*M**_r_* = 191.19	*D*_x_ = 1.359 Mg m^−^^3^
Orthorhombic, *P**c**a*2_1_	Mo *K*α radiation, λ = 0.71073 Å
Hall symbol: P 2c -2ac	θ = 2.6–25.0°
*a* = 10.0768 (7) Å	µ = 0.10 mm^−^^1^
*b* = 12.0387 (8) Å	*T* = 293 K
*c* = 7.7047 (6) Å	Needle, pale yellow
*V* = 934.67 (12) Å^3^	0.4 × 0.23 × 0.2 mm
*Z* = 4	

### Data collection

**Table:**

Bruker SMART CCD area-detector diffractometer	1436 independent reflections
Radiation source: fine-focus sealed tube	1189 reflections with ( > 2σ(*I*)
Graphite monochromator	*R*_int_ = 0.033
phi and ω scans	θ_max_ = 30.0°, θ_min_ = 1.7°
Absorption correction: multi-scan (*SADABS*; Bruker, 2001)	*h* = −13→14
*T*_min_ = 0.980, *T*_max_ = 0.984	*k* = −16→12
7118 measured reflections	*l* = −10→10

### Refinement

**Table:**

Refinement on *F*^2^	Primary atom site location: structure-invariant direct methods
Least-squares matrix: full	Secondary atom site location: difference Fourier map
*R*[*F*^2^ > 2σ(*F*^2^)] = 0.049	Hydrogen site location: inferred from neighbouring sites
*wR*(*F*^2^) = 0.145	H-atom parameters constrained
*S* = 0.83	*w* = 1/[σ^2^(*F*_o_^2^) + (0.1215*P*)^2^ + 0.160*P*] where *P* = (*F*_o_^2^ + 2*F*_c_^2^)/3
1436 reflections	(Δ/σ)_max_ < 0.001
128 parameters	Δρ_max_ = 0.33 e Å^−^^3^
1 restraint	Δρ_min_ = −0.22 e Å^−^^3^

### Special details

**Table:**

Geometry. All e.s.d.'s (except the e.s.d. in the dihedral angle between two l.s. planes) are estimated using the full covariance matrix. The cell e.s.d.'s are taken into account individually in the estimation of e.s.d.'s in distances, angles and torsion angles; correlations between e.s.d.'s in cell parameters are only used when they are defined by crystal symmetry. An approximate (isotropic) treatment of cell e.s.d.'s is used for estimating e.s.d.'s involving l.s. planes.
Refinement. Refinement of *F*^2^ against ALL reflections. The weighted *R*-factor *wR* and goodness of fit *S* are based on *F*^2^, conventional *R*-factors *R* are based on *F*, with *F* set to zero for negative *F*^2^. The threshold expression of *F*^2^ > σ(*F*^2^) is used only for calculating *R*-factors(gt) *etc*. and is not relevant to the choice of reflections for refinement. *R*-factors based on *F*^2^ are statistically about twice as large as those based on *F*, and *R*- factors based on ALL data will be even larger.

### Fractional atomic coordinates and isotropic or equivalent isotropic displacement parameters (Å^2^)

**Table:**

	*x*	*y*	*z*	*U*_iso_*/*U*_eq_	
C1	0.1765 (3)	−0.1284 (2)	−0.3293 (4)	0.0457 (6)	
H1	0.1447	−0.1956	−0.3719	0.055*	
C2	0.3054 (2)	−0.0942 (2)	−0.3241 (4)	0.0447 (6)	
H2	0.3770	−0.1355	−0.3647	0.054*	
C3	0.1905 (2)	0.03689 (18)	−0.2134 (3)	0.0364 (5)	
C4	0.1407 (2)	0.13759 (19)	−0.1392 (4)	0.0394 (5)	
H4	0.0487	0.1416	−0.1308	0.047*	
C5	0.20598 (19)	0.22724 (19)	−0.0798 (4)	0.0366 (4)	
C6	0.1238 (2)	0.32086 (19)	−0.0127 (4)	0.0416 (5)	
C7	0.1271 (3)	0.4938 (2)	0.1330 (5)	0.0623 (9)	
H7A	0.0624	0.5220	0.0509	0.075*	
H7B	0.0806	0.4701	0.2368	0.075*	
C8	0.2242 (4)	0.5808 (3)	0.1760 (6)	0.0696 (10)	
H8A	0.2917	0.5503	0.2499	0.104*	
H8B	0.1802	0.6406	0.2350	0.104*	
H8C	0.2641	0.6082	0.0713	0.104*	
C9	0.3471 (2)	0.23680 (19)	−0.0740 (4)	0.0421 (5)	
N1	0.10482 (18)	−0.04511 (16)	−0.2601 (3)	0.0420 (5)	
H1A	0.0200	−0.0440	−0.2477	0.050*	
N2	0.31525 (19)	0.00888 (17)	−0.2512 (3)	0.0396 (5)	
N3	0.4589 (2)	0.2480 (2)	−0.0668 (5)	0.0642 (8)	
O1	0.00530 (18)	0.32337 (16)	−0.0221 (4)	0.0626 (7)	
O2	0.19908 (17)	0.40053 (15)	0.0574 (3)	0.0515 (5)	

### Atomic displacement parameters (Å^2^)

**Table:**

	*U*^11^	*U*^22^	*U*^33^	*U*^12^	*U*^13^	*U*^23^
C1	0.0384 (11)	0.0307 (12)	0.0680 (15)	0.0007 (9)	−0.0019 (11)	−0.0053 (12)
C2	0.0371 (11)	0.0332 (12)	0.0637 (15)	0.0045 (9)	0.0035 (10)	−0.0040 (12)
C3	0.0309 (9)	0.0250 (10)	0.0533 (13)	−0.0030 (8)	0.0008 (8)	0.0027 (9)
C4	0.0323 (9)	0.0308 (11)	0.0551 (12)	−0.0001 (8)	0.0024 (10)	0.0029 (9)
C5	0.0348 (9)	0.0251 (9)	0.0500 (12)	0.0025 (7)	−0.0003 (9)	0.0015 (9)
C6	0.0371 (10)	0.0286 (11)	0.0591 (14)	−0.0003 (8)	0.0065 (10)	0.0011 (10)
C7	0.0554 (16)	0.0366 (14)	0.095 (2)	0.0014 (11)	0.0195 (16)	−0.0130 (15)
C8	0.086 (3)	0.0436 (15)	0.079 (2)	−0.0112 (15)	0.017 (2)	−0.0204 (16)
C9	0.0397 (11)	0.0264 (10)	0.0602 (14)	0.0028 (8)	−0.0018 (11)	−0.0017 (10)
N1	0.0291 (8)	0.0292 (9)	0.0677 (13)	−0.0027 (7)	0.0001 (9)	−0.0027 (10)
N2	0.0323 (9)	0.0297 (10)	0.0566 (11)	−0.0022 (7)	0.0014 (9)	0.0001 (8)
N3	0.0388 (10)	0.0502 (14)	0.103 (2)	0.0028 (9)	−0.0041 (14)	−0.0151 (15)
O1	0.0395 (9)	0.0418 (10)	0.1065 (19)	0.0051 (8)	0.0059 (10)	−0.0154 (11)
O2	0.0434 (9)	0.0291 (8)	0.0819 (15)	−0.0001 (6)	0.0067 (9)	−0.0126 (9)

### Geometric parameters (Å, º)

**Table:**

C1—N1	1.346 (3)	C6—O1	1.197 (3)
C1—C2	1.363 (3)	C6—O2	1.337 (3)
C1—H1	0.9300	C7—C8	1.472 (5)
C2—N2	1.366 (3)	C7—O2	1.458 (3)
C2—H2	0.9300	C7—H7A	0.9700
C3—N2	1.334 (3)	C7—H7B	0.9700
C3—N1	1.360 (3)	C8—H8A	0.9600
C3—C4	1.431 (3)	C8—H8B	0.9600
C4—C5	1.344 (3)	C8—H8C	0.9600
C4—H4	0.9300	C9—N3	1.135 (3)
C5—C9	1.428 (3)	N1—H1A	0.8600
C5—C6	1.491 (3)		
			
N1—C1—C2	105.9 (2)	C8—C7—O2	107.9 (3)
N1—C1—H1	127.0	C8—C7—H7A	110.1
C2—C1—H1	127.0	O2—C7—H7A	110.1
N2—C2—C1	110.8 (2)	C8—C7—H7B	110.1
N2—C2—H2	124.6	O2—C7—H7B	110.1
C1—C2—H2	124.6	H7A—C7—H7B	108.4
N2—C3—N1	110.9 (2)	C7—C8—H8A	109.5
N2—C3—C4	129.2 (2)	C7—C8—H8B	109.5
N1—C3—C4	119.9 (2)	H8A—C8—H8B	109.5
C5—C4—C3	130.1 (2)	C7—C8—H8C	109.5
C5—C4—H4	114.9	H8A—C8—H8C	109.5
C3—C4—H4	114.9	H8B—C8—H8C	109.5
C4—C5—C9	124.3 (2)	N3—C9—C5	177.6 (3)
C4—C5—C6	116.95 (19)	C1—N1—C3	107.74 (18)
C9—C5—C6	118.8 (2)	C1—N1—H1A	126.1
O1—C6—O2	124.9 (2)	C3—N1—H1A	126.1
O1—C6—C5	123.5 (2)	C3—N2—C2	104.54 (19)
O2—C6—C5	111.56 (19)	C6—O2—C7	115.6 (2)
			
N1—C1—C2—N2	0.6 (4)	C2—C1—N1—C3	−0.5 (3)
N2—C3—C4—C5	−4.1 (5)	N2—C3—N1—C1	0.2 (3)
N1—C3—C4—C5	177.9 (3)	C4—C3—N1—C1	178.6 (3)
C3—C4—C5—C9	−2.6 (5)	N1—C3—N2—C2	0.1 (3)
C3—C4—C5—C6	178.8 (3)	C4—C3—N2—C2	−178.1 (3)
C4—C5—C6—O1	−6.1 (5)	C1—C2—N2—C3	−0.4 (3)
C9—C5—C6—O1	175.1 (3)	O1—C6—O2—C7	2.7 (5)
C4—C5—C6—O2	174.2 (3)	C5—C6—O2—C7	−177.6 (3)
C9—C5—C6—O2	−4.5 (4)	C8—C7—O2—C6	−170.5 (3)

### Hydrogen-bond geometry (Å, º)

**Table:**

*D*—H···*A*	*D*—H	H···*A*	*D*···*A*	*D*—H···*A*
C4—H4···O1	0.93	2.38	2.771 (3)	105
N1—H1*A*···N2^i^	0.86	2.11	2.951 (3)	167
C1—H1···O1^ii^	0.93	2.45	3.328 (4)	158

Symmetry codes: (i) *x*−1/2, −*y*, *z*; (ii) −*x*, −*y*, *z*−1/2.
